# *Cis pallidus* Mellié, 1849: redescription, new synonym, geographic distribution, and host fungi records

**DOI:** 10.3897/zookeys.762.23433

**Published:** 2018-05-31

**Authors:** Paula V. Borlini, Cristiano Lopes-Andrade, Lucimar S. Araujo

**Affiliations:** 1 Programa de Pós-Graduação em Ecologia, Departamento de Biologia Geral, Universidade Federal de Viçosa, Campus Viçosa, Viçosa – MG, 36570-900, Brazil; 2 Laboratório de Sistemática e Biologia de Coleoptera, Departamento de Biologia Animal, Universidade Federal de Viçosa, Campus Viçosa, Viçosa – MG, 36570-900, Brazil; 3 Instituto de Ciências Biológicas e da Saúde, Universidade Federal de Viçosa, Campus Rio Paranaíba, MG 230, Km 8, Caixa Postal 22, Rio Paranaíba – MG, 38810-000, Brazil

**Keywords:** Ciinae, Ciini, minute tree-fungus beetles, Neotropical

## Abstract

*Cis pallidus* Mellié, 1849 is redescribed based on specimens from Northeast, Southeast, and South Brazil, and from Argentina. A lectotype is designated for *Cis semipallidus* Pic, 1916, and the species is synonymized with *C. pallidus*. The first host fungi records and a distribution map for the species are provided.

## Introduction

*Cis* Latreille is the most diverse genus of Ciidae with approximately 400 described species and a worldwide distribution (Oliveira et al. 2013, Lawrence 2016). The genus contains more than half of all described Ciidae species (Lawrence 1971, Oliveira et al. 2013, Lawrence 2016). The Neotropical species of *Cis* (biogeographic regions *sensu* Morrone 2015) are represented by nearly 70 described species and at least half of them are organized into artificial species-groups, such as the *comptus*, *creberrimus*, *melliei*, *pallidus*, *taurus*, *tricornis*, and *vitulus* species-groups.

The *pallidus*-group comprises *C. corticinus* Gorham, 1883 from Totonicapán, Guatemala, *C. pallidus* Mellié, 1849 from the state of Bahia, Northeast Brazil, *C. semipallidus* Pic, 1916 from Buenos Aires, Argentina, and *C. tetracentrum* Gorham, 1886, which occurs from southern California and Arizona to southern Mexico (Mellié 1849, Lawrence 1971, Gorham 1883, 1886,). These four species share an elongate body with single and uniform elytral punctation, dorsal vestiture of short to long bristles, slightly tumid prosternum, and males with a very small sex patch on the first abdominal ventrite or none at all (Lawrence 1971; pers. obs.). These species are morphologically closely related to species in the *vitulus*-group, which are similar but usually have a comparatively less elongate body (Lawrence 1971; pers. obs.).

The aim of this paper is to redescribe *C. pallidus*, propose a new synonym, and provide new host fungi and geographic distribution records.

## Materials and methods

The examined specimens are listed in the section on “type material” and “additional material” below. A total of 12 males from eight localities were dissected, as follows (number of specimens between parentheses): Buenos Aires (1; lectotype of *Cis semipallidus*) and Famaillá (1), in Argentina; Rio de Janeiro (1), São João del-Rei (1), Viçosa (5), Palotina (1), Nova Teutônia (1) and Urubici (1), in Brazil. Abdominal ventrites shown in Fig. 29 and genitalia in Figs 34–35 were extracted from the same male. Genitalia shown in Figs 30–31 were extracted from the male shown in Fig. 16; the same applies for Figs 43–44 and Fig. 20.

Museum abbreviations are as follows:

**ANIC** Australian National Insect Collection, CSIRO Entomology (Canberra, Australian Capital Territory, Australia)

**CELC** Coleção Entomológica do Laboratório de Sistemática e Biologia de Coleoptera da Universidade Federal de Viçosa (Viçosa, MG, Brazil)

**DZUP** Coleção Entomológica Pe. Jesus Santiago Moure, Universidade Federal do Paraná (Curitiba, PR, Brazil)

**FMNH** Field Museum of Natural History (Chicago, Illinois, USA)

**MACN** Museo Argentino de Ciencias Naturales “Bernardino Rivadavia” (Buenos Aires, Argentina)

**MCNZ** Fundação Zoobotânica do Rio Grande do Sul (Porto Alegre, RS, Brazil)

**MNHN** Muséum National d’Histoire Naturelle (Paris, France)

**MNRJ** Museu Nacional do Rio de Janeiro (Rio de Janeiro, RJ, Brazil)

Terms for external morphology and male terminalia of ciids follow Lopes-Andrade and Lawrence (2005, 2011), Lawrence et al. (2011), and Lawrence (2016), but see also Oliveira et al. (2013) for an explanation on the use of “tegmen”. The following abbreviations are used for measurements (in mm) and ratios:

**BW** (basal width of scutellar shield),

**CL** (length of antennal club measured from base of the eighth to apex of the tenth antennomere),

**EL** (elytral length along the midline),

**EW** (greatest width of both elytra),

**FL** (length of antennal funicle measured from base of the third to apex of the seventh antennomere),

**GD** (greatest depth of body measured in lateral view),

**GW** (greatest diameter of eye),

**PL** (pronotal length along midline),

**PW** (greatest pronotal width),

**SL** (length of scutellar shield),

**TL** (total length counted as EL+PL, *i.e.* excluding head).

The GD/EW and TL/EW ratios indicate the degree of body convexity and elongation, respectively.

A total of 21 males and 20 females were measured, with representative specimens from all examined localities. Measurements of antennomeres, GW, and BW provided in the description are the mean measurements of three males from three localities (Viçosa, Atílio Vivacqua, and Nova Teutônia); in these cases, standard deviations are not provided because they were 0.01 or less.

For scanning electron microscopy (SEM), specimens were dehydrated in a series of alcohol and acetone solutions, critical point dried (CPD 020, Balzers, Liechtenstein), mounted on aluminum stubs and sputter coated with gold (sputter module SCA 010, Balzers). Samples were then examined under a SEM (LEO VP 1430, Zeiss). Transcription of labels, dissection, photography under optical equipment, and measurement of specimens followed the methods provided by Araujo and Lopes-Andrade (2016). Names of host fungi extracted from labels were updated consulting the online database of Index Fungorum (http://www.indexfungorum.org) and are summarized in the section “Host fungi”. The criteria provided in Orledge and Reynolds (2005) are followed for determining breeding records. The distribution map (Fig. 45) was generated using the on-line SimpleMappr tool (Shorthouse 2010).

## Taxonomy

**Figures 1–8.**
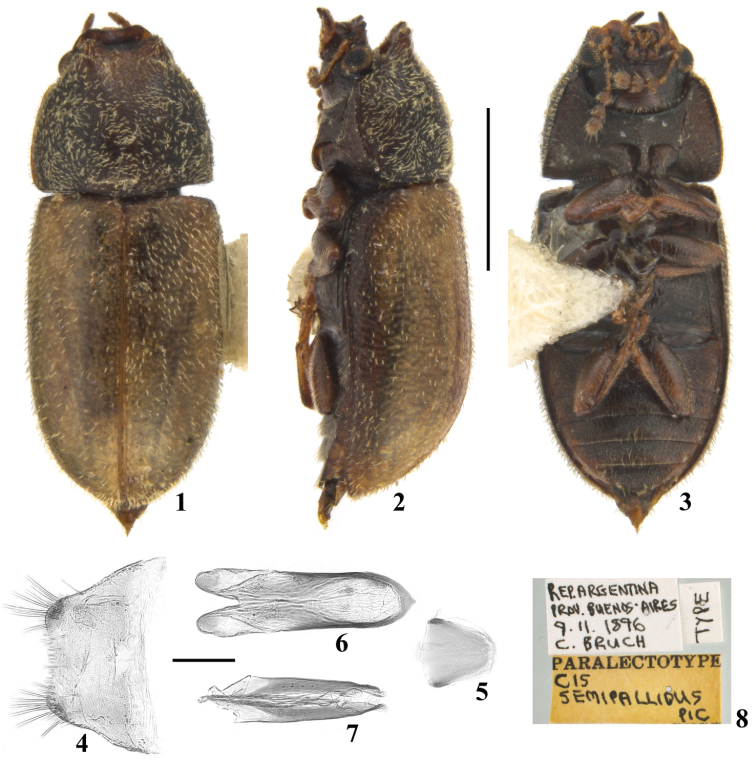
Male paralectotype of *Cis semipallidus* Pic, 1916. **1** Dorsal view **2** Lateral view **3** Ventral view **4** Sternite VIII **5** Basal piece **6** Tegmen **7** Penis **8** Labels. Scale bars: 1 mm (**1–3**); 0.1 mm (**4–7**).

**Figures 9–14.**
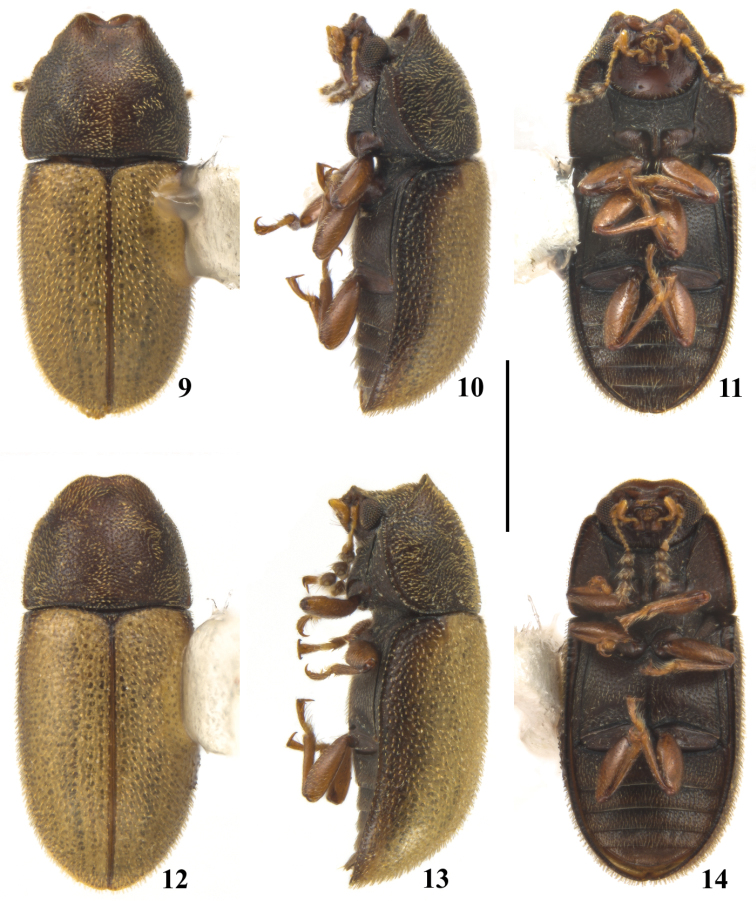
*Cis pallidus* Mellié, 1849, male (**9–11**) and female (**12–14**) from Viçosa, state of Minas Gerais, Southeast Brazil. **9, 12** Dorsal view **10, 13** Lateral view **11, 14** Ventral view. Scale bar: 1 mm.

**Figures 15–20.**
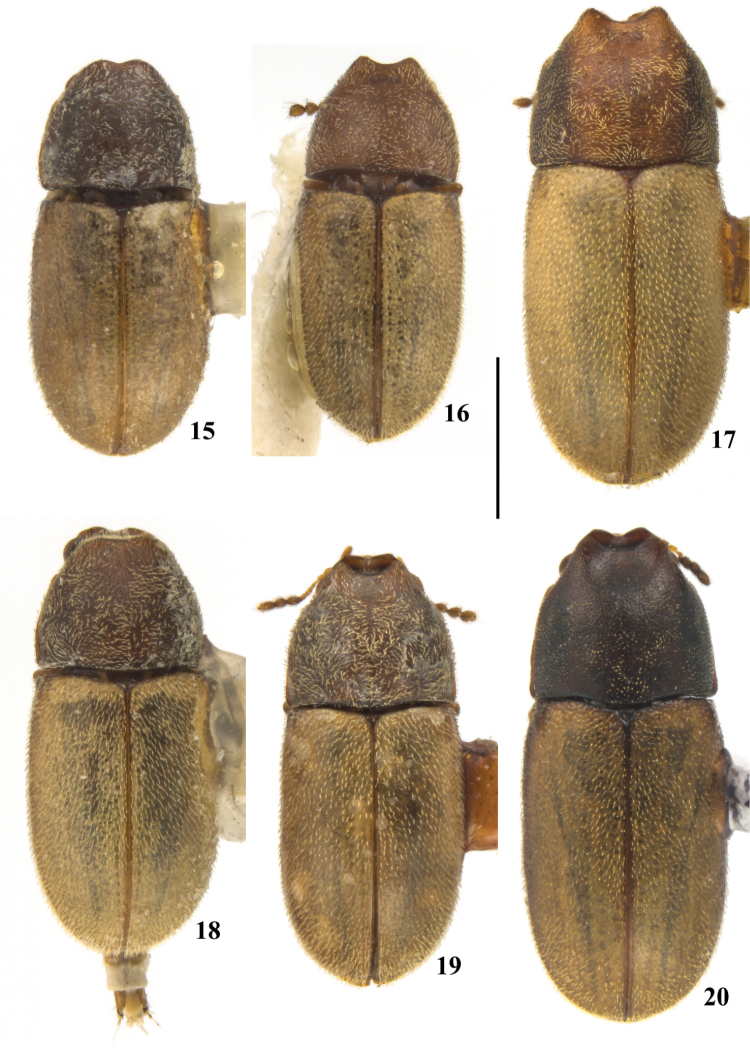
*Cis pallidus* Mellié, 1849, dorsal view from various localities, shown in the same scale. **15** Female from the state of Bahia, compared with the holotype deposited in the MNHN **16** Male from Corcovado, state of Rio de Janeiro **17** Male from Marechal Cândido Rondon, state of Paraná **18** Female from the province of Tucuman, Argentina **19** Male from Nova Teutônia, state of Santa Catarina **20** Male from Urubici, state of Santa Catarina. Scale bar: 1 mm.

**Figures 21–28.**
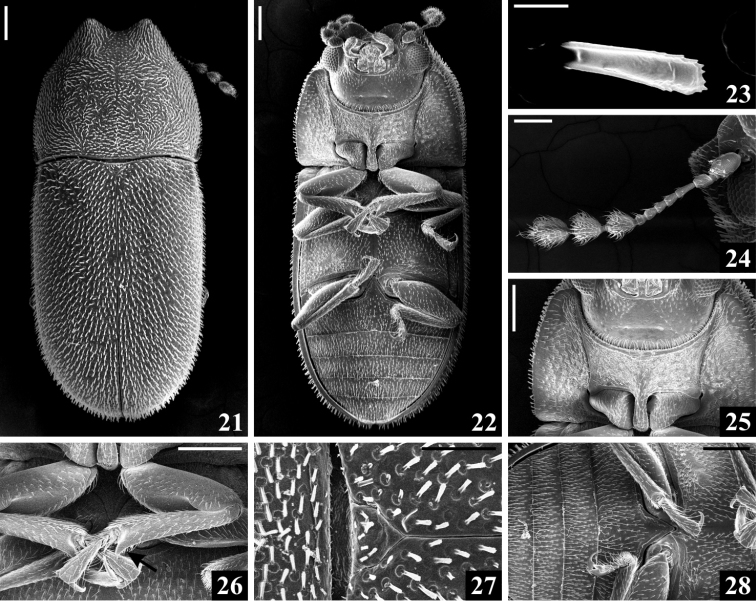
*Cis pallidus* Mellié, 1849, scanning electron microscopy. **21** Dorsal view **22** Ventral view **23** Elytral bristle **24** Antenna **25** Part of head and prothorax in ventral view **26** Protibiae, showing the outer apical angle (arrow) **27** Part of pronotum and elytra, with scutellar shield **28** Part of metaventrite and abdominal ventrites. Scale bars: 0.2 mm (**21–22, 25–26, 28**), 0.01 mm (**23**), 0.1 mm (**24, 27**).

Variation in pronotal and elytral color occurred between specimens from the same locality or even from the same basidiome. Also noticeable was variation in the length of dorsal bristles, mainly those on the pronotum (compare Figs 1, 9, 12, 15–21). The comparatively darker elytra and longer pronotal bristles of specimens of the type series of *C. semipallidus* were observed in several named specimens of *C. pallidus*.

**Figures 29–44.**
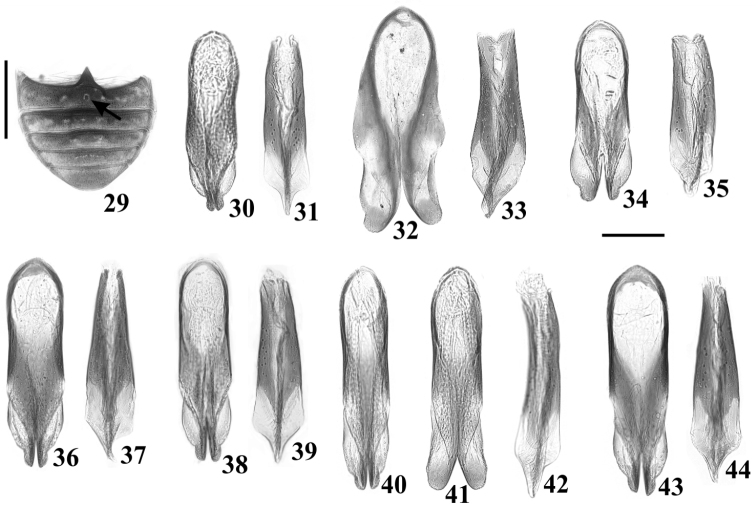
*Cis pallidus* Mellié, 1849. **29** Abdominal ventrites of a male from São João del-Rei, state of Minas Gerais, showing a small sex patch at the first ventrite (arrow), possibly vestigial **30–44** Tegmen (**30, 32, 34, 36, 38, 40–41, 43**) and penis (**31, 33, 35, 37, 39, 42, 44**) of males from various localities **30–31** Corcovado, state of Rio de Janeiro **32–33**Viçosa, state of Minas Gerais **34–35** São João del-Rei, state of Minas Gerais **36–37** Palotina, state of Paraná **38–39**Province of Tucuman, Argentina **40–42** Nova Teutônia, state of Santa Catarina, showing tegmen before (**40**) and after distortion (**41**) **43–44** Urubici, state of Santa Catarina. Scale bars: 0.5 mm (**29**); 0.1 mm (**30–44**).

There was little variation in size and morphology of tegmen and penis between dissected specimens from eight localities (Figs 6–7, 30–44). Length of tegmen varied from 0.29 to 0.36 mm, and of penis from 0.26 to 0.33 mm. The very rounded apical lobes of tegmen observed in two cases (Figs 6, 32) were artifacts of preparation, and occurred when the tegmen was dorso-ventrally-flattened between slide and cover slip. To confirm this, we photographed a tegmen before (Fig. 40) and after distortion (Fig. 41). No conspicuous sex patch was observed externally on the first abdominal ventrite of males, neither under stereomicroscopy nor under SEM (Figs 22, 28), but a possibly vestigial small mark was observed in all dissected males (e.g. Fig. 29, arrow). No diagnostic features to sustain *C. semipallidus* as a separate species have been found; thus we propose it as a junior synonym of *C. pallidus*.

### Cis pallidus

Taxon classificationAnimaliaColeopteraCiidae

Mellié, 1849

Figs 1–8 , 9–14 , 15–20 , 21–28 , 29–44 , 45

Cis pallidus Mellié, 1849: 246–247, pl. 10, fig. 10.
Cis semipallidus Pic, 1916: 5, **syn. n.**

#### Diagnosis.

Distinguished from other South American ciids by the elongate body (TL/EW at least 2), reddish to dark brown head and pronotum, yellowish elytra with a black band on both sides, and pronotum projected forward, partially or completely covering head when seen from above. Among South American ciid species with light-colored elytra, *Orthocis platensis* Brèthes, 1922 differs in the pronotum being not projected over the head. *Cis bisbidens* Gorham, 1883 differs in lacking lateral elytral band, in the developed projections associated with the male head and pronotum, and the small but conspicuous sex patch on first abdominal ventrite of the male. *Cis granarius* Mellié, 1849 has a comparatively stouter body and the sides of pronotum are light-colored. In *C. grossus* Mellié, 1849 and *C. validithorax* Pic, 1916 only the apical portion of the elytra is light-colored. *Cis steinheili* Reitter, 1878 is devoid of conspicuous lateral dark longitudinal band on elytra and the male has an obvious sex patch on the first abdominal ventrite.

#### Description of adult males.

***Body*** elongate, subparallel-sided; head, pronotum and scutellar shield reddish-brown (Fig. 16) to dark brown (Fig. 20); pronotum sometimes lighter on disc than on sides (Fig. 17); elytral disc mostly pale yellowish (Figs 9, 17) to yellowish brown (Fig. 1), slightly translucent, gradually darkening to sides and forming a distinct dark longitudinal band on both sides easily seen in lateral view (Figs 2, 10); ventral surface (Figs 3, 11) dark brown, with appendages usually lighter; dorsal vestiture of short decumbent (Fig. 23), goldish bristles, as long as puncture-width (Fig. 20) and up to three puncture-widths (Fig. 19), the same length or longer on elytra than on pronotum, usually sparser on elytra than on pronotum; ventral vestiture of decumbent whitish, slender setae, longest on metaventrite and abdominal ventrites. ***Head*** partially or completely covered by anterior pronotal plate when seen from above (Figs 1, 9, 16–17, 19–20); dorsal surface with small shallow punctures and microreticulate interspaces; anterocephalic edge weakly projected forward and upward, sinuous, forming two short, rounded projections (Fig. 11), separated from each other by about one-fifth the basal width of scutellar shield. ***Antennae*** (Fig. 24) bearing 10 antennomeres, with measurements as follow (n = 3, mean; in mm): 0.09, 0.05, 0.05, 0.03, 0.03, 0.03, 0.02, 0.08, 0.08, 0.09 (FL 0.16; CL 0.26; CL/FL 1.56). ***Eyes*** coarsely facetted, with approx. 100 ommatidia; GW (n = 3, mean; in mm) 0.17. ***Pronotum*** with coarse punctures, separated from each other by one puncture-width or less; interspaces of punctures, microreticulate; anterior edge produced forward overhead, broadly emarginate at apex, forming two short subtriangular plates (Figs 1, 9, 16–17, 19–20); anterior portion with concave impression before anterior plates; anterior angles produced forward and broadly rounded; lateral edges finely crenulate, barely visible when seen from above. ***Scutellar shield*** (Fig. 27) subpentagonal, with few punctures and bristles; BW (n = 3, mean; in mm) 0.11. ***Elytra*** with single sized punctation, punctures shallower, finer and sparser than those on pronotum; interspaces of punctures irregular close to anterior portion of elytra and smooth at disc. ***Metathoracic wings*** developed, apparently functional. ***Hypomera*** with shallow, inconspicuous punctation; interspaces, finely shagreened. ***Prosternum*** (Fig. 25) slightly tumid at midline; surface similar to that of hypomera. ***Prosternal process*** (Fig. 25) about as long as prosternal disc, curved, slightly enlarged close to rounded apex. ***Protibiae*** slightly expanded to apex; outer apical angle acute (Fig. 26, arrow); apical edge devoid of spines. ***Meso- and metatibiae*** comparatively less expanded than protibiae; outer apical angle rounded; apical edge with row of minute spines. ***Metaventrite*** with punctation denser than those on hypomera, more conspicuous close to sides; interspaces shagreened; discrimen shallow, about half the length of metaventrite at midline. ***Abdominal ventrites*** with punctation and surface similar to that of metaventrite; length of ventrites (measured in male from Viçosa; in mm, from base to apex at longitudinal midline) as follows: 0.3, 0.12, 0.11, 0.11, 0.13; first ventrite apparently devoid of sex patch (Figs 22, 28), but with small barely discernible mark in slide preparation (Fig. 29, arrow). ***Male abdominal terminalia*** with ***sternite VIII*** (Fig. 4) subtrapezoidal, posterior edge slightly curved inwardly and with short setae, sides rounded and bearing long slender setae. ***Tegmen*** (Figs 6, 30, 32, 34, 36, 38, 40–41, 43) subcylindrical, 2.89–4.45× as long as wide, 1–1.13× as long as penis; apical portion with short emargination, forming two lateral lobes; apex narrow, preceded by lateral, broadly rounded membranous flaps. ***Basal piece*** (Fig. 5) subtriangular, about as long as wide, 1/3 to 1/4 length of tegmen. ***Penis*** (Figs 7, 31, 33, 35, 37, 39, 42, 44) subcylindrical, 3.6–4.35× as long as wide, gradually expanding from base to apex, apex subtriangular.

**Females**. Projections of head and pronotum more rounded and less prominent than in males. Otherwise like males, but first abdominal ventrite devoid of any discernible mark. ***Female abdominal terminalia*** (in specimen from Viçosa with everted terminalia) with ***paraprocts*** 1.25× as long as gonocoxites; each ***gonocoxite*** with three ventral lobes; ***gonostyli*** inserted at top of gonocoxites.

#### Measurements and ratios.

Males, measurements in mm (n = 21): TL 1.95–3.00 (2.47 ± 0.32), PL 0.70–1.00 (0.86 ± 0.09), PW 0.80–1.20 (0.99 ± 0.12), EL 1.20–2 (1.60 ± 0.24), EW 0.85–1.30 (1.07 ± 0.13), GD 0.60–1.15 (0.83 ± 0.13), PL/PW 0.72–1.12 (0.87 ± 0.10), EL/EW 1.26–1.94 (1.49 ± 0.13), EL/PL 1.38–2.05 (1.86 ± 0.17), GD/EW 0.54–1.00 (0.77 ± 0.09), TL/EW 2.04–3.00 (2.30 ± 0.20). Females, measurements in mm (n = 20): TL 2.15–3.05 (2.53 ± 0.23), PL 0.75–1.00 (0.85 ± 0.07), PW 0.85–1.20 (1.02 ± 0.08), EL 1.40–2.05 (1.68 ± 0.17), EW 0.95–1.35 (1.11 ± 0.10), GD 0.75–1.05 (0.86 ± 0.09), PL/PW 0.75–0.89 (0.83 ± 0.03), EL/EW 1.42–1.56 (1.50 ± 0.03), EL/PL 1.76–2.20 (1.97 ± 0.11) GD/EW 0.68–0.87 (0.76 ± 0.04), TL/EW 2.16–2.36 (2.26 ± 0.04).

#### Type material.

♀ **Holotype** “Cis pallidus Mel. Bahia. T. Cast. 72 [green disc, handwritten] \ Bahia. Mocquerys [handwritten] \ Cis pallidus Reiche [handwritten] \ LECTOTYPE [printed] Cis pallidus Mellié [handwritten]”; ♂ **lectotype of *Cis semipallidus* Pic, 1916, here designated** (MNHN) “Rep Argentina. Prov. Buenos Aires 9.11.1896 C. Bruch \ Cis près Cis bisbidens Gorh. [handwritten] \ Cis [handwritten] \ Type [yellow paper; handwritten] \ C. semipallidus Pic [handwritten] \ Type [red paper] \ LECTOTYPE [printed] Cis semipallidus Pic [handwritten]; 1 ♂ (MNHN, dissected) “Rep. Argentina Prov. Buenos Aires 9.11.1896 C. Bruch [handwritten] \ Type [handwritten] \ PARALECTOTYPE [printed] Cis semipallidus Pic [handwritten]”.

#### Additional material.

**BRASIL**: 1 ♀ (MNHN) “Museum Paris Brésil Bahia (Tabacs) A. Grouvelle 1913\ Cis pallidus Mellié 1969 J. F. Lawrence”; 1 ♂ and 1 ♀ (CELC); “BRASIL: ES, Atílio Vivacqua, Mata do Zé [sic], 16.vi.2007, Furieri, K. S. & Ana Paula”; 1 ♂ and 2 ♀(MNRJ) “Saude 29.vi.[19]14 \ MNRJ 230”; 1 ♂ (DZUP, dissected) “Coleção M. Alvarenga \ CORCOVADO Guanabara BRASIL x.1968 Alvarenga & Seabra \ Cis pallidus Mellié, 1849 C. Lopes-Andrade det. \ DZUP 273653”; 22 ♂ (CELC, 5 dissected) and 13 ♀ (CELC) “BRASIL: MG, Viçosa, “Atrás do Insetário” 18.xi.2003 legs. D. J. Souza & C. Lopes-Andrade \ ex *Trametes hirsuta*”; 61 specimens (CELC, in alcohol) “Brasil: MG, Viçosa, Bom Jesus, 13.vii.2006 Oliveira, C. B.”; 19 specimens (CELC, in alcohol) “Brasil: MG, Viçosa, Apiário 09.vi.2010 leg. L. A. O. Campos”; 4 ♂ (CELC) and 3 ♀ (CELC) “BRASIL: MG, Viçosa, “Violeira” 17.xii.2004 leg. A. A. Zacaro”; 5 ♂ and 6 ♀ (CELC) “BRASIL: MG, Viçosa, Mata da Biologia, 03.v.2014, Lopes-Andrade et al. leg”; 2 ♀ (CELC) “BRASIL: MG, Jequeri; Piscamba 09.vi.2009 leg. D. M. da Silva”; 6 ♂ (CELC, 1 dissected) and 6 ♀ (CELC) “BRASIL: MG, São João del Rei [sic] Colônia 25.xii.2011 De Oliveira, G. A. & De Oliveira, E. H.”;13 ♀ (CELC) “BRASIL: MG, Juiz de Fora Campus UFJF 11.vii.2012 Pecci-Maddalena leg.”; 2 ♂ and 2 ♀ (CELC) “BRASIL: MG, Ibitipoca, 03.iii.2014. leg. Pecci-Maddalena, I. \ *Trametes hirsuta*”; 2 ♂ (1 DZUP; 1 CELC, dissected) “BRASIL: PR, Palotina Parque Estadual de São Camilo (coleta manual) 02.iii.2011 G. A. Oliveira, col. \ Ciidae sp. 17 C. S. Santos, det. 2011 \ Coletado no fungo: *Trametes* sp. (Polyporaceae) V. G. Cortez det. 2011 \ C342 \ Cis pallidus Mellié, 1849 C. Lopes-Andrade det.”; 1 ♂ (ANIC, dissected) and 1 ♀(ANIC) “Nova Teutonia Santa Catarina BRAZIL March F. Plaumann”; 1 ♂(ANIC) “BRAZIL: Santa Catarina, Nova Teutonia Dec. F. Plaumann”; 1 ♂ (ANIC) “Brasilien [Marechal Cândido] Rondon 24° 38’B. 54° 07’ L F. Plaumann”; 1 ♀(ANIC) “Santa Catarina BRAZIL xi.64 Fritz Plaumann”; 1 ♀ (DZUP) “Brasilien Nova Teutonia 27° 11’ B 52° 23’ L 300-500m xii.1980 Fritz Plaumann \ Cis pallidus Mellié, 1849 C. Lopes-Andrade det. \ DZUP 273657”; 1 ♂ (FMNH) “Nova Teutonia, Sta. Catarina, BRAZ. Fritz Plaumann leg. \ Cis pallidus Mell. det. J. F. Lawrence \ FMNH 261”; 3 ♀ (FMNH) “Nova Teutonia, Sta. Catarina, BRAZ. 5.viii.44 Fritz Plaumann leg. \ Cis pallidus Mell. det. J. F. Lawrence \ FMNH 259; 260; 262”; 3 ♂ (CELC, 1 dissected) and 1 ♀ (CELC) “BRASIL: SC, Urubici (Estrada para Serra do Corvo) 06.iii.2011 Grossi & Parizotto”; 1 ♂ (MCNZ) “Torres, RS 21/XI/1976 [handwritten] A. Lise leg. [p.] COL. NCN 28.253 [handwritten]”; 1 ♀ (ANIC) “Brazil J. Rick \ J. F. Lawrence Lot.1995 \ *Polyporus polyzonus* [=*Trametes polyzona*]”. **ARGENTINA**: 4 ♀ (FMNH) “R. Argentina La Plata Col. C. Bruch\ FMNH 263”; 1 ♂ (ANIC, dissected) and 1 ♀ (ANIC) “ARG: Tucuman Famailla vi.11.1971 \ L. A. Stange Lot 13 \ *Coriolus pinistus* [sic] [=*Trametes hirsuta*]”; 1 ♂ and 1 ♀ (MACN) on the same card “Rep Argentina. Prov. Buenos Aires 9.11.1896 C. Bruch \ Cis près bisbidens Gorh. [handwritten]”.

#### Comments.

**Figure 45.**
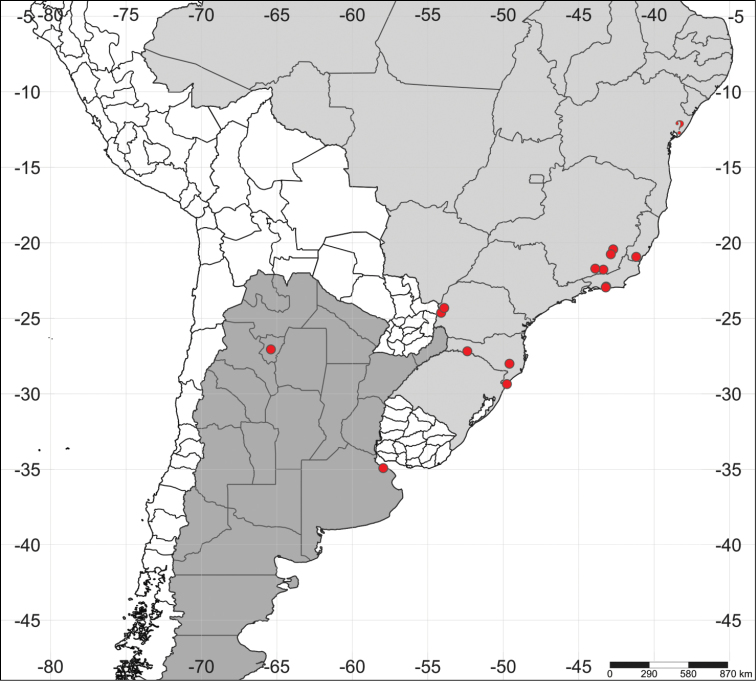
Distribution map of *Cis pallidus* Mellié, 1849. The question mark shows a presumed locality of the species in Recôncavo Baiano, an area of old tobacco farms in the state of Bahia. See text for more details.

There was no consistent difference in the morphology of tegmen and penis between specimens with different dorsal coloration and length of dorsal bristles, and such variation occurred within the populations throughout the geographic extension of the species. Therefore, we consider that the abovementioned specimens all belong to a single species. After the description of *C. pallidus*, Mellié (1849: 247) wrote “Provient de Bahia; a été donné à M. Reiche par M. Mocquerys de Rouen”, a statement in the singular, suggesting that he had only one specimen at the time of the description. It is important to note that Mellié, in the same work, clearly used plural in case he had examined two or more specimens. Therefore, we consider the single type specimen located in the MNHN as the holotype, even though it has a lectotype label. Two specimens from MACN, pinned at the same card, have the same locality label of the type series of *C. semipallidus*, but these do not have any indication whether they were examined or not by Maurice Pic. Thus we think they do not belong to the type series but they were possibly collected together. The specimen from the MNHN labeled “(…) Bahia (Tabacs) A. Grouvelle (…)” is possibly from Recôncavo Baiano (Fig. 45, question mark), a name for the geographic area around Bay of All Saints, the biggest bay at the northeastern coast of Brazil. The collector Antoine Grouvelle was the director of the “Manufactures nationales des Tabacs” (national manufacturers of tobacco) and used to catch small insects in the tobacco leaves exported to France. These insects, mostly Coleoptera, were probably retained during their flight by the more or less abundant pubescence and viscosity which covers the tobacco leaves; it is also possible that some of them had been attracted by the rainwater which remains in the axils of the leaves, and that others came from the washing water in the country of production (Régimbart 1895). In the 19^th^ century, most of the tobacco production from Bahia came from Recôncavo Baiano; therefore, we consider that it is plausible that this specimen of *C. pallidus* has come from this area.

#### Host fungi.

*Trametes hirsuta* Lloyde (Polyporaceae), one record and two breeding records; *Trametes polyzona* Pers. (Polyporaceae), one record; and *Trametes* sp. (Polyporaceae), one record.

## Supplementary Material

XML Treatment for Cis pallidus
